# How low should we target the LDL goal to improve survival for acute coronary syndrome patients in Hong Kong?

**DOI:** 10.1186/s12872-015-0117-y

**Published:** 2015-10-07

**Authors:** Vivian W. Lee, Raymond Y. Chau, Herich Y. Cheung, Cheuk Man Yu, Yat Yin Lam, Bryan P. Yan

**Affiliations:** School of Pharmacy, Faculty of Medicine, The Chinese University of Hong Kong, 8th Floor, Lo Kwee-Seong Integrated Biomedical Sciences Building, Area 39, Shatin, Hong Kong; Department of Medicine and Therapeutics, Faculty of Medicine, The Chinese University of Hong Kong, Shatin, Hong Kong

**Keywords:** Lipid-lowering, Myocardial Infarction, Hong Kong, Clinical impact, Statin, Lipid Management

## Abstract

**Background:**

Utilization of lipid-lowering agents has been associated with improved long-term outcomes in acute coronary syndrome (ACS) patients. However, updated data regarding local use and outcomes was lacking.

**Methods:**

We retrospectively reviewed 696 hospitalized patients in the local ACS registry of Prince of Wales Hospital during 1 January 2008 to 31 December 2009 with data retrieved using computerized clinical records of all patients.

**Results:**

Among the 402 MI patients included, 104 (25.9 %) were not prescribed with statins at discharge. Percutaneous coronary intervention (PCI) not performed or planned during hospitalization (OR: 0.324, *p* = 0.001) and latest lower LDL-C level before discharge (OR: 0.221 for an increment of 1 mmol/L, *p* = 0.009) were significant independent predictors of the absence of statin prescriptions at discharge. A significantly lower all-cause mortality rate (14.4 % vs 51.7 %, *p* < 0.001), fewer total hospitalizations (*p* < 0.001) and fewer hospitalizations due to cardiovascular problems (*p* < 0.001) were observed in patients discharged with statins. LDL-C goal attainment of < 2.6 mmol/L resulted in a significant reduction in mortality (10.8 % vs 24.2 %, *p* = 0.001), but not for goal attainment of < 1.8 mmol/L. Significant difference in survival existed only when LDL-C cut-off values were above 2.4 mmol/L.

**Conclusions:**

This study revealed the under-utilization of statin therapy in eligible MI patients at discharge and unsatisfactory percentages of LDL-C goal attainment, and also reassured the role of low LDL-C reduction to < 2.6 mmol/L in the management of MI. However, the current study did not show that the lower LDL-C reduction improved survival of ACS patients. Further research should be conducted to assess the necessity of aggressive LDL-C reduction to < 1.8 mmol/L in local patients.

## Background

Encompassing unstable angina, non–ST-segment elevation myocardial infarction (NSTEMI) and ST-segment elevation myocardial infarction (STEMI), acute coronary syndrome (ACS) is a more severe type of coronary heart disease (CHD) attributed to the rupture of atherosclerotic plaque present in the coronary artery. Serum cholesterol levels have been shown to continuously correlate with CHD risk over a broad range of cholesterol values [1–3], and elevated levels of low-density lipoprotein cholesterol (LDL-C) are thought to be a key determinant of CHD risk [4]. Utilization of lipid-lowering agents, particularly 3-hydroxy-3-methylglutaryl coenzyme A reductase inhibitors (statins), have been associated with improved long-term outcomes in ACS patients [5–9], and initiation of statins has been recommended by international guidelines as a secondary preventive strategy for recurrent ischemia and mortality in ACS patients [10–14]. Current guidelines recommend aggressive LDL-C reduction in patients with established CHD [10–14]. The new ACC/AHA adult treatment panel recommends more than 50 % reduction of the current LDL-C levels for high risk patients [13]. A previous local study conducted in the early 2000’s revealed that 44 % of patients being put on statins failed to achieve their cholesterol goals by a median follow-up period of 1.9 years [15]. Furthermore, most clinical trials evaluating benefits of lipid-lowering therapy were mainly carried out in Caucasian populations and had included few Chinese subjects. However, differences in the pattern of lipid abnormalities and their relative impact on CHD risk have been observed among ethnic groups [16, 17]. Since both cholesterol levels and CHD mortality are perceived to be lower in Chinese population when compared to Western populations [18–20], the necessity for aggressive LDL-C reduction in Chinese population remains uncertain. Therefore, the current study primarily aimed to assess the current prescribing pattern of lipid-lowering agents and the percentage of LDL-C goal attainment in myocardial infarction (MI) patients in local practice, and to evaluate clinical outcomes of patients stratified by prescription of statins and by LDL-C level attained after discharge. Our study also aimed to examine the effect of aggressive LDL-C reduction on survival in local MI patients, and hence to evaluate its necessity.

## Methods

This retrospective study utilized clinical data retrieved from computerized clinical records of all MI patients who were admitted to the Prince of Wales Hospital, a teaching and major acute public hospital in Hong Kong, during the period of 1 January 2008 to 31 December 2009. The current study was conducted prior to the 2013 ACC/AHA guidelines. Personal particulars of patients were extracted from the ACS registry, a local registry that records all patients admitted to the hospital due to an ACS event. Our study only included patients with ischemic symptoms or electrocardiographic changes accompanied by an elevation of cardiac troponin T to > 0.10 ng/mL and with a valid LDL-C levels. Relevant clinical information was extracted up to the starting date of data collection, 18 December 2011. The protocol of this study was approved by the Joint CUHK-NTEC Clinical Research Ethics Committee.

Statins were the primary lipid-lowering agents being investigated in our study. Patients with combination therapy of lipid-lowering agents or non-statin therapy were excluded in the analysis. The agent prescribed and its dose at various time points including the day before admission, during hospitalization, at discharge and at first follow-up were collected for analysis of prescribing pattern in local clinical practice. For various statins being used, their daily doses were expressed as the equivalent dose with respect to simvastatin 20 mg [21, 22]. The dose equivalence was based on a 25–30 % reduction in LDL-C [21].

Demographic information, smoking status, medical history and co-morbidities (heart failure – Patients with positive cardiac echocardiography findings and confirmed diagnosis by cardiologists; hypertension – Patients with at least 2 consecutive blood pressure measurements that exceed the target range according to the Joint National Committee VII Report on hypertension; diabetes – Patients with hemoglobin A1C greater than 7 %), presence of ST-segment elevation in electrocardiogram, history of interventional strategies performed or planned during hospitalization, medication profiles and blood lipid profiles before and during index hospitalization, and serum levels of alanine aminotransferase (ALT) and creatine kinase (CK) during index hospitalization were recorded for the analysis of patient characteristics.

Date of death, lipid profiles after index hospitalization, number of hospitalizations after index hospitalization, and history of revascularization after index hospitalization were also collected for the analysis of patient outcomes. Only unscheduled admissions were counted towards post-discharge hospitalizations; scheduled workups, surgeries and ward follow-ups were excluded. Post-discharge hospitalizations fell into the cardiac category if patients were admitted due to cardiovascular events secondary to any previous ACS episode (such as arrhythmia, shortness of breath or fluid overload secondary to heart failure, cerebrovascular accident (CVA), acute pulmonary edema, cardiac chest pain and another ACS episode). Follow-up period of an individual, as defined by the duration of post-discharge survival period till the date of 18 December 2011, was also calculated.

### Statistical method

The analyzing cohort was divided into 2 sub-groups based on whether statins were prescribed at discharge for the comparison of patient characteristics and outcomes. LDL-C attainment analysis was done with similar parameters by the stratification of the cohort with averaged lipid values after 6 months post-discharge into groups based on 2 different LDL-C goal attainments of 2.6 mmol/L and 1.8 mmol/L respectively. Hazard ratios with 95 % confidence intervals were also presented. Logistic regression analyses were performed to evaluate the influence of patient characteristics on prescription of statins at discharge while Cox regression analysis was performed to evaluate the influence of patient characteristics on mortality; variables with *p* ≤ 0.20 in univariate analysis were encompassed in the multivariate model.

Survival probabilities of the analyzed population were illustrated using the Kaplan-Meier survival curves with stratifications based on prescription of statins at discharge and attainment of the two LDL-C goals (<1.8 mmol/L and < 2.6 mmol/L). Survival probabilities of the whole analyzed population stratified by various post-discharge LDL-C cut-off values were also illustrated. Hazard ratios (HRs) with 95 % confidence intervals (95 % CI) were also presented.

Continuous variables were analyzed using the Wilcoxon rank sum test and expressed as medians with interquartile ranges, while categorical variables were analyzed using the Pearson chi-square test and expressed as frequencies with percentages. Trend test was performed for number of hospitalizations post-discharge and number of revascularizations. Difference in survival probabilities was analyzed by the log-rank test. A *p*-value of < 0.05 was considered to be statistically significant for the tests. All analyses were performed using SPSS version 19.0.

## Results

**Fig. 1:**
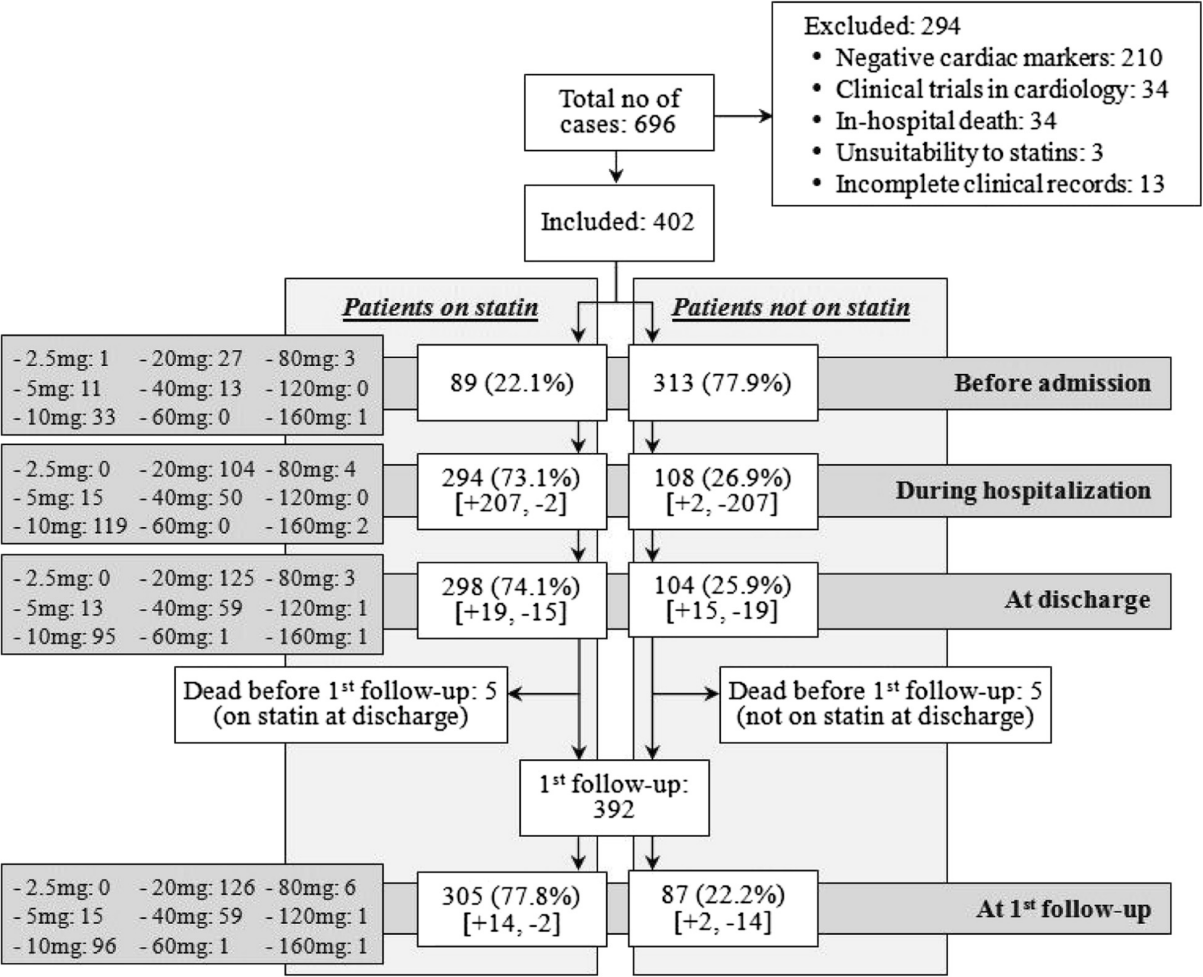
Number of patients being prescribed with statins at various time points. *The dose listed were the equivalent daily dose with respect to simvastatin

We retrospectively reviewed 696 patients with MI, and 402 of which were included in the analysis (Fig. 1). It is important to note that 3 patients were excluded from data analysis due to unsuitability for statins therapy because of elevation of liver function tests or elevation of creatinine kinase with complain of myalgia because of statin use at baseline. We did not detect any side effect related to statins during the follow up period in our study subjects who were on statin therapy. Among them, 191 (47.5 %) had a traceable record of a complete lipid panel before admission, and 110 (57.6 %) of which had a LDL-C ≥ 2.6 mmol/L. Not all patients had at least one LDL-C check-ups during hospitalization, and only 340 (84.6 %) had so (Table 1). Although a total of 209 (61.5 %) patients had LDL-C ≥ 2.6 mmol/L during hospitalization, 30 (14.4 %) of them were not prescribed any statins at discharge (Table 1). By taking LDL-C < 1.8 mmol/L as the more aggressive goal for MI patients, 307 (90.3 %) patients were not at goal before discharge, and 63 (20.5 %) of which did not receive any statin therapy at discharge. Excluding patients died within the 6-month period post-discharge (*n* = 22), a total of 346 (91.1 %) patients had at least one lipid panel conducted 6 months after discharge (Table 1). The use of statins at discharge was significantly associated with lower LDL-C level 6-month post-discharge (*p* < 0.001), although there were more patients with LDL between 1.8 mmol/L and 2.6 mmol/L than less than 1.8 mmol/L.

Patients were stratified into statin and non-statin groups for investigation of patient characteristics associated with the absence of statin prescription at discharge. Among those patients discharged with statins (*n* = 298), majority of our patients were on monotherapy lipid lowering agent (mainly statin) at discharge. There were 268 (90 %) patients who were prescribed with simvastatin, Rosuvastatin, atorvastatin and lovastatin were prescribed in 24 (8.1 %), 5 (1.7 %) and 1 (0.3 %) patients respectively. We excluded patients with combination therapy of lipid-lowering agents or non-statin therapy. There were 6 patients being excluded: 3 patients on monotherapy gemfibrozil 1200 mg/day, 1 patient with gemfibrozil 600 mg/day plus simvastatin 40 mg, 2 patients with ezetimibe 10 mg/day plus atorvastatin 80 mg. Variables with *p* ≤ 0.20 in univariate analysis were encompassed in the multivariate model (Table 2). The multivariate Cox regression analysis revealed that PCI not performed or planned during hospitalization (OR: 0.324, *p* = 0.001) and latest lower LDL-C level before discharge (OR: 0.221 for an increment of 1 mmol/L, *p* = 0.009) were significant independent predictors for the absence of statin prescription (Table 2). In other words, patients without PCI performed or planned during hospitalization and with low LDL-C level before discharge were less likely to be prescribed with statins.

**Table 1:** LDL-C levels and statin utilization of patients at various time points

		LDL-C ≤ 1.8	1.8 < LDL-C ≤2.6	LDL-C > 2.6	*P* value
Before admission	Statin group (*n* = 130)^a^	15 (11.5 %)	37 (28.5 %)	78 (60.0 %)	0.203
	Non-statin group (*n* = 61)^a^	13 (21.3 %)	16 (26.2 %)	32 (52.5 %)	
	Total (*n* = 191)	28 (14.7 %)	53 (27.7 %)	110 (57.6 %)	/
Latest during hospitalization	Statin group (*n* = 269)^a^	25 (9.3 %)	65 (24.2 %)	179 (66.5 %)	<0.001
	Non-statin group (*n* = 71)^a^	8 (11.3 %)	33 (46.5 %)	30 (42.3 %)	
	Total (*n* = 340)	33 (9.7 %)	98 (28.8 %)	209 (61.%)	/
Half-year post-discharge	Statin group (*n* = 272)^a^	98 (36.0 %)	115 (42.3 %)	59 (21.7 %)	<0.001
	Non-statin group (*n* = 61)^a^	15 (20.3 %)	23 (31.1 %)	36 (48.6 %)	
	Total (*n* = 191)	113 (32.7 %)	138 (39.9 %)	95 (27.5 %)	/

^a^Patients were stratified according to prescription of statins at discharge

**Fig. 2:**
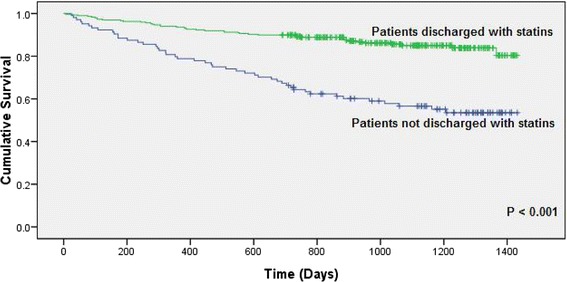
Kaplan-Meier survival function by prescription of statins at discharge.Note: All-cause mortality = 43/298 (14.4%) in statin group versus 46/104 (51.7%) in non-statin group

More patients prescribed with statins at discharge achieved a lower LDL-C level 6-month post-discharge (*p* < 0.001). In a medium follow-up period of 1022 (752.5–1236.0) days in all patients, a significantly lower all-cause mortality rate was observed in patients discharged with statins (14.4 % vs 51.7 %, *p* < 0.001). Furthermore, patients discharged with statins were found to have a lower number of total hospitalizations and hospitalizations due to cardiovascular problems secondary to any previous ACS episode after index hospitalization. Patients with statins who had at least one hospitalization due to cardiovascular disease were 116 (38.9 %) versus 58 (55.8 %) in the non-statin group [*p* < 0.001]. Nevertheless, percentages of patients requiring revascularizations including PCI (statin group: *n* = 30 [10.1 %]; non-statin group: *n* = 5 [4.8 %]; *p* = 0.133) and CABG (statin group: *n* = 5 [1.7 %]; non-statin group: *n* = 2 [1.9 %]; *p* = 0.869) were similar between the statin and non-statin cohorts. Kaplan-Meier curves illustrating survival of patients discharged with or without statins also revealed that prescription of statins at discharge was significantly associated with reduction of mortality (HR: 0.285, 95 % CI: 0.188–0.433, *p* < 0.001) (Fig. 2). We found that there were statistically significant differences between the statin group versus non-statin group in terms of baseline characteristics. The patients who did not take statins at discharge were older (average age: 70 years old versus 79 years old; *p* < 0.001), with more previous ischemic heart disease/acute coronary syndrome (19.8 % versus 32.7 %; *p* = 0.007), cerebrovascular accident (8.4 % versus 14.4 %; *p* = 0.077), heart failure (9.4 % versus 30.8 %; *p* < 0.001) and poorer creatinine kinase levels (3.0 % versus 8.9 %; *p* = 0.023) as well as liver function (0.7 % versus 3.9 %; *p* = 0.041). Therefore, although the patients who did not take statins may have achieved the LDL-C goal, they had poorer clinical outcomes due to other co-existing conditions.

Patient characteristics of the cohort of 346 subjects with at least one lipid panel checked 6 months post-discharge were evaluated. Several patient characteristics were associated with a higher probability of achieving a desirable LDL-C 6 months after discharge. When LDL-C < 2.6 mmol/L was taken as the desirable level, these characteristics included being prescribed with statins at discharge (*p* < 0.001) and a lower LDL-C (*p* = 0.028) and TG (*p* = 0.043) before discharge. However, if the more stringent LDL-C goal of 1.8 mmol/L was taken into consideration, characteristics associated with higher probability of achieving a desirable 6-month post-discharge LDL-C level included a higher age (*p* = 0.040), being not an active smoker (*p* = 0.002), being prescribed with statins at discharge (*p* = 0.010) and a lower LDL-C before discharge (*p* < 0.001).

With a LDL-C < 2.6 mmol/L attained 6 months after discharge, a lower all-cause mortality was observed (10.8 % vs 24.2 %, *p* = 0.001). However, this difference became insignificant if the more stringent goal of 1.8 mmol/L was used (13.3 % vs 15.0 %, *p* = 0.947). Moreover, no significant association could be noted between attainment of LDL-C goal of either < 2.6 mmol/L or < 1.8 mmol/L and number of hospitalizations as well as revascularization. Survival of patients by attainment of LDL-C goals of < 2.6 mmol/L and < 1.8 mmol/L also revealed that attainment of LDL-C < 2.6 mmol/L was associated with reduction of mortality significantly (HR: 0.413, 95 % CI: 0.237–0.720, *p* = 0.002), but not attainment of LDL-C < 1.8 mmol/L (HR: 0.891, 95 % CI: 0.487–1.633, *p* = 0.710).

The multivariate Cox regression analysis encompassing variables with p ≤0.20 on univariate Cox regression analysis revealed that age at discharge (HR: 1.066 for an 1-year increment, *p* = 0.001), co-morbidity of heart failure (HR: 2.859, *p* = 0.002), STEMI at index hospitalization (HR: 0.318, *p* = 0.002) and first LDL-C level 6 months after discharge (HR: 1.824, *p* = 0.003 for an increment of 1 mmol/L) were significant predictors of prognosis (Table 3). Significant association was demonstrated between attainment of LDL-C goal of < 2.6 mmol/L and reduction in mortality, but not for attainment of LDL-C goal of < 1.8 mmol/L.

**Table 2:** Predictors of the absence of statin prescription in univariate and multiple logistic regression

Variable	Odds ratio	95 % CI	*p*-value
Univariate Logistic Regression Analysis			
Male sex	0.492	0.310–0.780	0.003
Age at discharge	1.059	1.036–1.083	<0.001
Active smoking status	0.642	0.366–1.124	0.121
Past medical history of IHD or ACS	1.968	1.195–3.241	0.008
Past medical history of CVA	1.840	0.929–3.645	0.080
Co-morbidity of hypertension	1.061	0.668–1.685	0.802
Co-morbidity of heart failure	4.286	2.424–7.578	<0.001
Co-morbidity of diabetes mellitus	1.459	0.930–2.289	0.101
STEMI at index hospitalization	0.616	0.389–0.975	0.038
PCI done or planned during index hospitalization	0.191	0.114–0.321	<0.001
CABG done or planned during index hospitalization	0.418	0.142–1.229	0.113
Latest TG level before discharge	0.682	0.485–0.960	0.028
Latest HDL-C level before discharge	1.270	0.590–2.736	0.541
Latest LDL-C level before discharge	0.551	0.398–0.763	<0.001
Latest serum ALT level before discharge > 3 × ULN	5.919	1.068–32.812	0.042
Latest serum CK level before discharge > 2 × ULN	3.141	1.211–8.419	0.019
Multiple Logistic Regression Analysis			
Male sex	0.829	0.429–1.604	0.578
Age at discharge	1.012	0.983–1.041	0.426
Active smoking status	1.337	0.626–2.857	0.453
Past medical history of IHD or ACS	1.200	0.569–2.531	0.633
Past medical history of CVA	0.643	0.230–1.794	0.399
Co-morbidity of heart failure	1.750	0.767–3.991	0.184
Co-morbidity of diabetes mellitus	1.258	0.686–2.309	0.458
STEMI at index hospitalization	0.753	0.417–1.358	0.346
PCI done or planned during index hospitalization	0.324	0.167–0.629	0.001
CABG done or planned during index hospitalization	0.458	0.162–1.824	0.324
Latest TG level before discharge	0.009	0.601–1.258	0.458
Latest LDL-C level before discharge	0.221	0.423–0.885	0.009
Latest serum ALT level before discharge > 3 × ULN	0.197	0.472–25.654	0.221
Latest serum CK level before discharge > 2 × ULN	0.950	0.660–7.462	0.197

**Table 3:** Predictors of mortality in univariate Cox proportional hazards regression model and multivariate Cox regression analysis

Variable	Hazard ratio	95 % CI	*p*-value
Univariate Cox proportional hazards regression model of mortality			
Male sex	0.525	0.346–0.796	0.002
Age at discharge	1.069	1.045–1.092	<0.001
Active smoking status	0.511	0.284–0.920	0.025
Past medical history of IHD or ACS	1.827	1.177–2.836	0.007
Past medical history of CVA	2.755	1.688–4.498	<0.001
Co-morbidity of hypertension	1.601	1.008–2.542	0.046
Co-morbidity of heart failure	4.266	2.770–6.570	<0.001
Co-morbidity of diabetes mellitus	1.786	1.174–2.715	0.007
STEMI at index hospitalization	0.345	0.213–0.560	<0.001
PCI done or planned during index hospitalization	0.185	0.108–0.318	<0.001
CABG done or planned during index hospitalization	0.609	0.223–1.660	0.332
Latest TG level before discharge	0.842	0.617–1.149	0.279
Latest HDL-C level before discharge	0.674	0.298–1.521	0.342
Latest LDL-C level before discharge	0.873	0.649–1.175	0.370
Prescription of statins at discharge	0.285	0.188–0.433	<0.001
Latest CK level before discharge > 2 × ULN	1.659	0.724–3.803	0.231
Latest serum ALT level before discharge > 3 × ULN	6.491	2.371–17.770	<0.001
First TG level 6 months after discharge	0.740	0.478–1.145	0.177
First HDL-C level 6 months after discharge	0.875	0.402–1.905	0.737
First LDL-C level 6 months after discharge	1.685	1.230–2.308	0.001
Number of in-patient hospitalization due to cardiac reasons	1.076	1.034–1.120	<0.001
Predictors of mortality in multivariate Cox regression analysis			
Male sex	0.936	0.487–1.801	0.843
Age at discharge	1.066	1.027–1.107	0.001
Active smoking status	1.602	0.671–3.825	0.289
Past medical history of IHD or ACS	0.493	0.237–1.023	0.057
Past medical history of CVA	1.804	0.866–3.758	0.115
Co-morbidity of hypertension	0.765	0.364–1.608	0.480
Co-morbidity of heart failure	2.859	1.448–5.646	0.002
Co-morbidity of diabetes mellitus	1.217	0.632–2.342	0.556
STEMI at index hospitalization	0.318	0.157–0.645	0.002
PCI done or planned during index hospitalization	0.468	0.203–1.075	0.074
Prescription of statins at discharge	0.833	0.416–1.664	0.604
Latest serum ALT level before discharge > 3 × ULN	0.961	0.113–8.116	0.971
First TG level 6 months after discharge	0.695	0.384–1.260	0.231
First LDL-C level 6 months after discharge	1.824	1.233–2.697	0.003
Number of in-patient hospitalization due to cardiac reasons	1.010	0.928–1.100	0.814

**Fig. 3:**
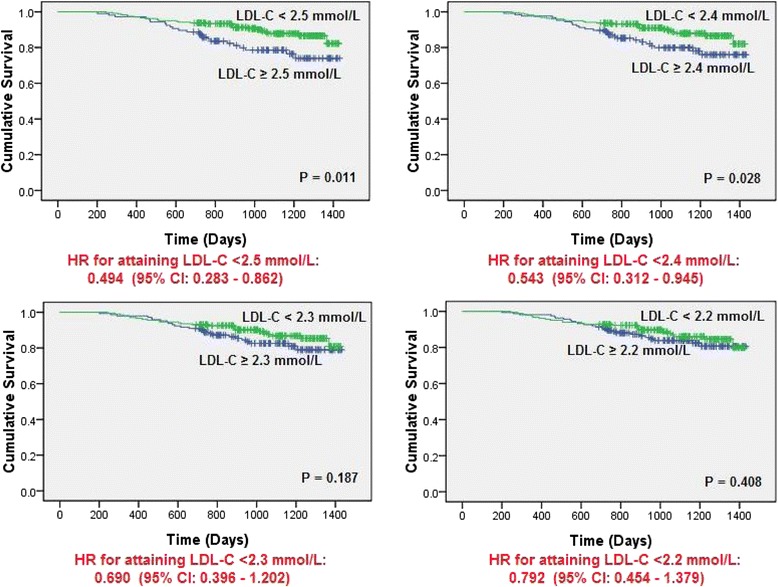
Kaplan-Meier survival function of all patients stratified by new LDL-C cut-off values

Stratifications of all patients for mortality analyses based on different LDL-C cut-off values produced survival curves with different significant levels (Fig. 3). A decrease in the survival difference between patients above and below cut-off values was observed when the LDL-C cut-off value became more aggressive from 2.6 mmol/L (*p* = 0.001) to 2.2 mmol/L (*p* = 0.408). Taking LDL-C level of 2.4 mmol/L as the cut-off, patients attaining LDL-C < 2.4 mmol/L at 6-month post-discharge had a significantly better survival than those did not (*p* = 0.028). However, this difference became insignificant when the LDL-C level of 2.3 mmol/L was taken as the cut-off (*p* = 0.187).

## Discussion

Lipid-lowering therapy including statin therapy is an important ACS management strategy to prevent complications secondary to ACS and all patients with baseline LDL-C not at goal should have lipid-lowering therapy for LDL-C goal attainment [10–14]. Our study, however, revealed the under-utilization of statin therapy in eligible STEMI and NSTEMI patients without any contraindication to statins and failure in LDL-C goal attainment in some patients.

A common approach for enhancing goal achievement involves the use of higher equipotency doses of statins such as titrating up simvastatin doses or utilizing statins with higher equipotency than simvastatin, the use of add-on agents such as ezetimibe to statins [23–25] and to establish the role of pharmacists in lipid attainment [26, 27]. A prospective controlled study showed that patient compliance and ATP III LDL-C goal attainment could be significantly enhanced compared with control group after individualized counseling and follow-up by pharmacists [26].

Prescription of statins was significantly associated with lower mortality (*p* < 0.001), which is in line with other studies [5–9]. Significantly much lower cardiac and all-cause re-hospitalizations were also observed with the use of statins at discharge, but not for revascularization. Patients with statins in this study had significantly higher percentages of in-hospitalization PCI (done or planned), suggesting more severe forms of ACS and hence, more aggressive treatments such as revascularization may be preferred over medication treatment alone in case of recurrent ACS. Willingness and appropriateness (such as age) of patients to undergo revascularization were also potential confounders.

Several patient characteristics have been identified to be associated with poorer prognosis in our study, which included older age, co-morbidity of heart failure, presentation of NSTEMI at index hospitalization and high LDL-C level at the first lipid panel at least 6-month after discharge. The presence of heart failure may reflect that myocardial cells are more severely damaged, which probably explains its association with higher mortality. Since poor control of blood pressure and blood glucose are significant risk factors of heart failure [28, 29], modifications of diet and medications targeting better control may be beneficial in delaying disease progression and thus reducing mortality. The association between high post-discharge LDL-C level and poor prognosis observed has illustrated that attainment of LDL-C goals is a more important predictor of death, but not prescription of statins. This has signified the importance of regular monitoring of blood lipid levels, with intensification of lipid-lowering therapy in patients not achieving desirable LDL-C levels. The association of non–ST-segment elevation with poorer prognosis seems to be surprising, as STEMI has been traditionally classified as the most severe manifestation of the ACS spectrum [12–14]. However, our finding is in fact consistent with previous retrospectively study that NSTEMI patients have unadjusted mortality rates higher than patients with STEMI [30–32]. Allen and his colleagues have shown that ST-elevation would be associated with the highest risk when potential confounders in baseline characteristics and treatment were adjusted, and indicated that NSTEMI patients are less likely to receive standard therapies because STEMI can be diagnosed immediately on electrocardiogram and has historically been viewed as the most concerning diagnosis in the spectrum of ACS [30].

With a significantly better survival observed in patients attaining levels of LDL-C below 2.6 mmol/L but not below 1.8 mmol/L, we have further investigated the survival probability of patients attaining different LDL-C levels post-discharge. Contrasting to current evidence that suggest the reduction of LDL-C to < 1.8 mmol/L in high risk patients [11], our results indicated that patients attaining levels between 1.8 mmol/L and 2.6 mmol/L had a better survival than those with levels below 1.8 mmol/L in our study. Given the differences may exist in the pattern of lipid abnormalities and their relative impact on CHD risk among ethnic groups, our results have created controversy on the necessity of aggressive reduction of LDL-C to below 1.8 mmol/L in local Chinese population. When more survival curves were plotted with stratification of the study population by various LDL-C cut-off values between 2.6 mmol/L and 1.8 mmol/L, a reduction in survival difference between patients below and above the cut-off value was observed as more aggressive LDL-C cut-off values were taken. A significant better survival was seen in patients attaining LDL-C < 2.4 mmol/L (when compared to those did not), but not in patients attaining LDL-C < 2.3 mmol/L (when compared to those did not). Therefore, our results suggested that the LDL-C goal in local population may be < 2.4 mmol/L, if a difference in threshold should exist among ethnic groups. The current 2013 ACC/AHA cholesterol guidelines recommended high-risk cardiovascular patients to attain 50 % LDL-C reduction^11^. In our current project, we observed that over 42.4 % of patients had a baseline LDL-C level of < 2.6 mmol/L before admission. Therefore, the target goal will be < 1.3 mmol/L according to the 2013 ACC/AHA cholesterol guidelines. According to our current study, the lower LDL-C reduction did not translate into better clinical survival. The cost of management may also increase due to the use of high-potency statin such as rosuvastatin that may pose economic concerns for healthcare providers.

Several limitations exist in our current study. Study population was restricted to patients admitted to a major teaching hospital in Hong Kong, in which clinical experience of physicians there may be different from that of other hospitals in Hong Kong. Therefore, the results obtained may not truly reflect the actual prescribing practice in Hong Kong. The multivariate Cox proportional hazards regression model of mortality was unable to include all variables that may possibly be associated with all-cause mortality observed during the follow-up duration, such as co-morbidities of fatal diseases and prescription of other evidence-based medicines in the management of MI. Last but not least, being a retrospective study analyzing computerized clinical records, the current study is unable to identify any factors that were not documented in computer records but may potentially influence the prescription of statins. More importantly, the possible existence of confounders in our study may have potentially caused the insignificant difference observed in survival between patients above and below LDL-C cut-off values when LDL-C levels below 2.3 mmol/L were taken as the cut-off values. In addition, we did not adjust for the patients’ characteristics fort he Kaplan Meier analysis due to small sample size. Further research, preferably randomized controlled trials, should be conducted to confirm our findings regarding the necessity of aggressive LDL-C reduction in local Chinese population.

## Conclusions

This study revealed the under-utilization of statin therapy in eligible MI patients at discharge, particularly in patients without undergoing PCI and with low LDL-C levels during hospitalization. Unsatisfactory LDL-C goal attainment in MI patients was observed. This study also reassured the role of LDL-C reduction to < 2.6 mmol/L in the management of MI. However, the current study did not show that the lower LDL-C reduction improved survival of ACS patients. Further research should be conducted to assess the necessity of aggressive LDL-C reduction to < 1.8 mmol/L in local patients.
